# Longitudinal measurement of serum neurofilament light in presymptomatic familial Alzheimer’s disease

**DOI:** 10.1186/s13195-019-0472-5

**Published:** 2019-02-20

**Authors:** Philip S. J. Weston, Teresa Poole, Antoinette O’Connor, Amanda Heslegrave, Natalie S. Ryan, Yuying Liang, Ronald Druyeh, Simon Mead, Kaj Blennow, Jonathan M. Schott, Chris Frost, Henrik Zetterberg, Nick C. Fox

**Affiliations:** 1 Dementia Research Centre, Department of Neurodegenerative Diseases, UCL Institute of Neurology, Queen Square, Box 16, London, WC1N 3BG UK; 2 Department of Medical Statistics, London School of Hygiene & Tropical Medicine, London, UK; 3 The UK Dementia Research Institute at UCL and Department of Neurodegenerative Diseases, UCL Institute of Neurology, London, UK; 4 MRC Prion Unit at UCL, Institute of Prion Diseases, London, UK; 5 Clinical Neurochemistry Laboratory, Institute of Neuroscience and Physiology, The Sahlgrenska Academy at the University of Gothenburg, Mölndal, Sweden

**Keywords:** Search terms, [26] Alzheimer’s disease, [91] autosomal dominant, [111] neurofilament light, [111] blood, [111] longitudinal

## Abstract

**Background:**

To investigate how serum neurofilament light (NfL) concentration changes through the course of disease in familial Alzheimer’s disease (FAD) and to assess when NfL concentration first increases.

**Methods:**

NfL was measured using an ultrasensitive immunoassay in 117 serum samples from 61 individuals from families with *PSEN1* or *APP* mutations in a longitudinal study (mean ± SD = 1.9 ± 1.1 visits/patient; inter-visit interval = 1.8 ± 1.1 years). The relationship between NfL concentration and estimated years to/from symptom onset (EYO) was modelled using linear regression, including all time points and robust standard errors to allow for repeated measurements, adjusting for age at visit and sex. Also, for the 27 participants who became symptomatic (during or before the study), NfL concentration was also modelled against known actual years to/from onset (AYO).

**Results:**

There were 15 non-carriers and 46 mutation carriers (21 symptomatic; 25 presymptomatic). NfL concentration was increased (*p* = 0.045) in mutation carriers compared with non-carriers 15 years prior to expected symptom onset, increasing progressively thereafter. There was a significant inter- and intra-individual variability in the longitudinal pattern of change. Modelling NfL for the 27 mutation carriers with known AYO also showed a progressive increase over time.

**Conclusions:**

There is evidence that serum NfL is increased more than a decade before the onset of clinical symptoms in FAD and rises thereafter. While there is variability in change over time, both within and between individuals, and more work is needed to understand the sources of this variability, serum NfL remains a promising, accessible biomarker of early neurodegeneration in presymptomatic Alzheimer’s disease.

**Electronic supplementary material:**

The online version of this article (10.1186/s13195-019-0472-5) contains supplementary material, which is available to authorized users.

## Background

Biomarkers sensitive to early neurodegeneration in Alzheimer’s disease (AD) can facilitate early diagnosis, monitor disease progression, and support trials of potential disease-modifying therapies. A blood-based biomarker would be less invasive and likely cheaper than currently available imaging and cerebrospinal fluid measures. We previously reported that the concentration of serum neurofilament light (NfL) in autosomal dominant familial Alzheimer’s disease (FAD) mutation carriers was increased prior to the onset of clinical symptoms [1]. Serum NfL has also been shown to be closely associated with validated imaging and cognitive measures of early neurodegeneration [1, 2].

However, it remains uncertain when in relation to symptom onset, serum NfL first increases. Also, while clinically important phenotypic differences have been found within the spectrum of FAD, particularly between amyloid precursor protein (*APP*) and presenilin 1 (*PSEN1*) mutation carriers, and within the *PSEN1* gene between mutations located pre and post-codon 200 [3], it is unknown whether this heterogeneity translates to differences in NfL concentration.

We therefore carried out a longitudinal study, with an increased sample size, of serum NfL in FAD mutation carriers and mutation-negative relatives.

## Methods

### Participants

We recruited 61 participants from 30 FAD families to a longitudinal study of FAD at the Dementia Research Centre, University College London, between April 2010 and February 2018. Eligibility was either (i) clinical diagnosis of FAD or (ii) an FAD-affected parent. Twenty-one participants were symptomatic, with pathogenic mutations in the *PSEN1* or *APP* genes; 40 were asymptomatic but at 50% risk of an inherited mutation and thereby of developing symptoms at a similar age to their parent.

Genetic test data on the presence or absence of a mutation in ‘at-risk’ participants were provided only to study statisticians. Estimated years to/from symptom onset (EYO) were calculated by subtracting parental age of onset of progressive cognitive symptoms from the participant’s current age. At each study visit, procedures undertaken included blood sampling, a semi-structured health questionnaire (including exclusion of recent head injury), neurological examination, and the clinical dementia rating scale.

In total, 117 serum samples were obtained and one third of participants (20/61) had three or more time points (mean ± SD per participant 1.92 ± 1.08). The mean ± SD inter-visit interval was 1.75 ± 1.09 years. An ultrasensitive immunoassay on the Simoa platform measured serum NfL, using a methodology described previously [4]. All samples were processed according to the same standardised operating procedure and measured as duplicates. Samples from all time points were also processed concurrently using the same kit and batch of reagents to maximise consistency of analyses. Coefficients of variation (CVs) for duplicate concentrations of subject samples and quality control (QC) samples were < 15%.

Following genetic testing, we found that there were a total of 46 mutation carriers (21 symptomatic and 25 presymptomatic) and 15 non-carriers in the study. The total number of serum samples from each group was 29 from the symptomatic mutation carriers, 54 from the presymptomatic carriers, and 36 from the non-carriers.

### Statistical analysis

Summary baseline descriptive data were calculated for symptomatic mutation carriers, presymptomatic mutation carriers, and non-carriers, and observed NfL concentrations plotted for carriers and non-carriers. For several participants, there were large within-person changes in NfL concentrations over relatively short time intervals. This meant that although we had longitudinal data, we have not analysed it in a way that explicitly models longitudinal change within subjects because the large within-person variability in observed rates of change created convergence problems when we tried to fit a mixed effects model. Instead, a linear regression model was used, including all time points and robust (Huber-White) standard errors that allowed for repeated measurements within individuals [5, 6]. The dependent variable (NfL) was log-transformed, and the relationship with EYO was modelled using genetic mutation status and linear, quadratic and cubic terms for EYO, plus their interactions with mutation status, as predictor variables, adjusting for age at visit and sex. The estimated geometric mean longitudinal NfL concentration profiles for the two groups (and 95% confidence intervals) were plotted against EYO standardising to the mean age and gender mix in the sample. We estimated the difference in geometric mean NfL for values of EYO between − 20 and 10 years, adjusted for age at visit and sex, and tested for when the difference between carriers and non-carriers was statistically significantly different from zero (*p* < 0.05).

Separately, for 27 participants who became symptomatic (before or during the study), NfL concentration was plotted against actual years to/from onset (AYO), rather than EYO. Non-carriers do not have AYO values, so for comparison, we used their mean serum NfL concentration ± 1.96SD to provide a 95% reference range), calculated on the log scale and back-transformed. For the 27 symptomatic carriers, NfL was modelled against AYO, using linear regression with robust standard errors as above; the quadratic and cubic terms for AYO were not statistically significant so were dropped, with only the linear term for AYO being significant. Non-carriers were not included in the AYO model. The modelled geometric mean longitudinal NfL concentration profile (and its 95% confidence interval) for symptomatic mutation carriers was plotted against AYO, with the same standardisation for age and gender as in the EYO graphs for consistency. In this case for comparison with non-carriers, we used the non-carrier geometric mean serum NfL concentration and its 95% confidence interval.

Separate models compared mean log NfL in *PSEN1* versus *APP* carriers and, in *PSEN1* carriers, compared those with mutations pre- versus post-codon 200. These models adjusted for age at visit, sex, and EYO, and utilised robust standard errors. All analyses used Stata v15 (StataCorp, College Station, TX, USA).

## Results

**Fig. 1:**
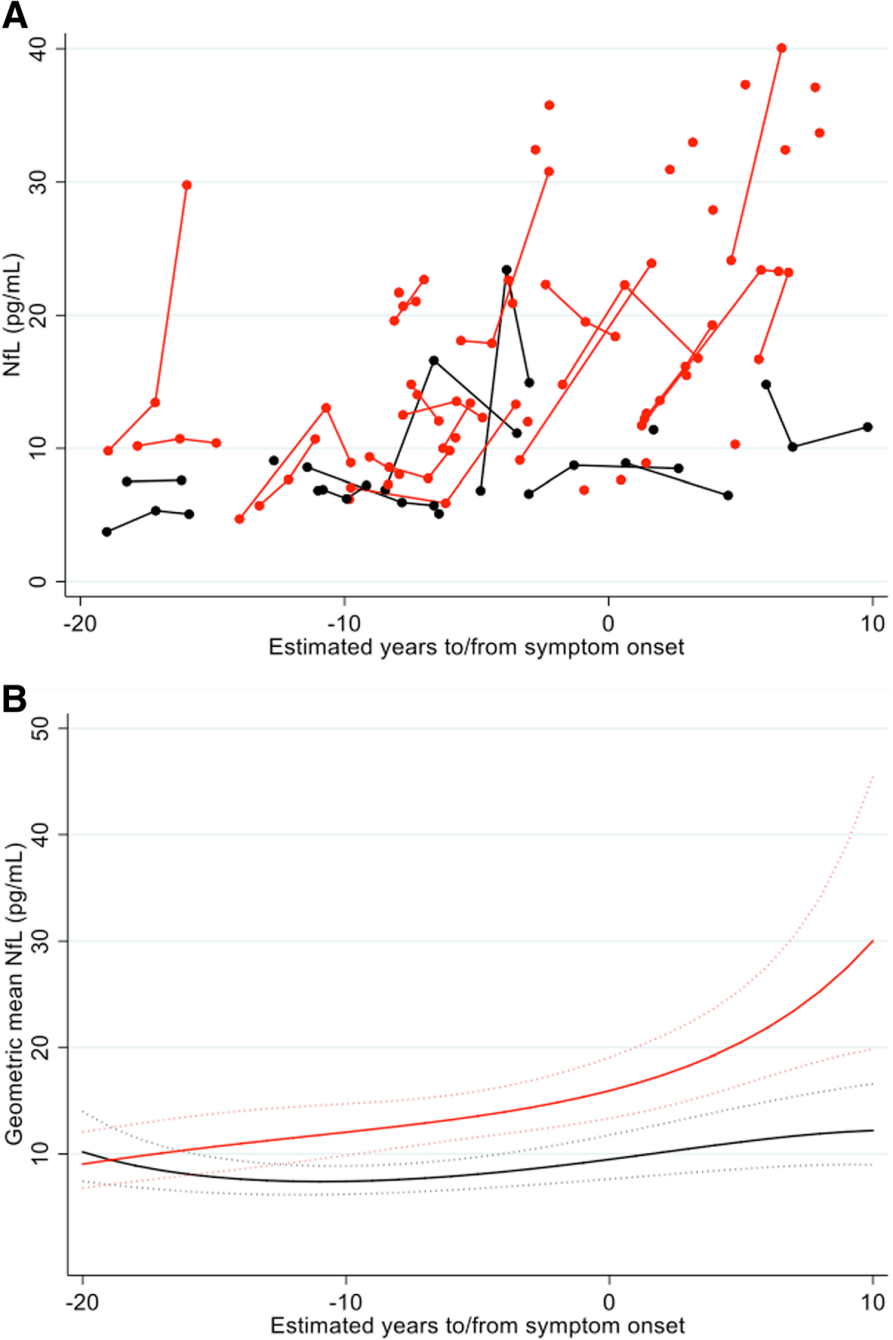
Serum NfL against EYO. Mutation carriers are represented in red and non-carriers in black. **a** Measured values of serum NfL concentration against EYO. Those measurements that belong to the same individual are connected by a line. To ensure it is not possible to identify any of the individual asymptomatic participants (based on their EYO) and so determine their mutation status, five outlying participants have been removed and a jitter of up to ± 2 years has been applied to all remaining participants. Also, for the three individuals who donated more than three samples, only their first three measurements are shown. **b** Geometric mean NfL modelled against EYO, for someone of average age (40.9 years) and sex (half male/female). Dotted lines indicate 95% confidence intervals. EYO estimated years to/from symptom onset. The geometric mean is the exponential of a mean calculated on the log scale

Participants’ demographic details and serum NfL values are in Table 1 and Fig. 1a. For mutation carriers, 37 had mutations in *PSEN1* and nine in *APP* (Additional file 1: Table S1).

**Table 1:** Participant demographics and serum NfL concentration. For variables with missing data points, the number of observations is shown underneath the group average value (e.g. *n* = *x*). *EYO* estimated years to/from onset, *MMSE* Mini-Mental State Examination, *CDR* Clinical Dementia Rating Scale, *SOB* sum of boxes

	Non-carriers *N* = 15	Presymptomatic *N* = 25	Symptomatic *N* = 21
Gender (female/male)	11/4	13/12	7/14
Age (mean, SD)	39.7 (9.1)	36.2 (5.8)	47.4 (9.3)
EYO (years) (mean, SD)	n/a	− 9.25 (6.21)	3.09 (3.53)
MMSE (median, IQR)	30 (30–30)	30 (29–30)	23 (19.5–26.5) (*n* = 20)
CDR Global (median, IQR)	0 (0–0)	0 (0–0)	0.5 (0.5–1.0) (n = 18)
CDR SOB (median, IQR)	0 (0–0)	0 (0–0)	3.25 (1.5–4.5) (*n* = 18)
Baseline NfL (pg/mL) (mean, SD)	10.34 (7.62)	12.83 (6.71)	22.60 (12.28)
Annualised rate of change in NfL (pg/mL/years) (mean, SD)*	0.51 (1.54) (*n* = 9)	0.67 (3.72) (*n* = 17)	3.52 (3.72) (*n* = 5)

**Fig. 2:**
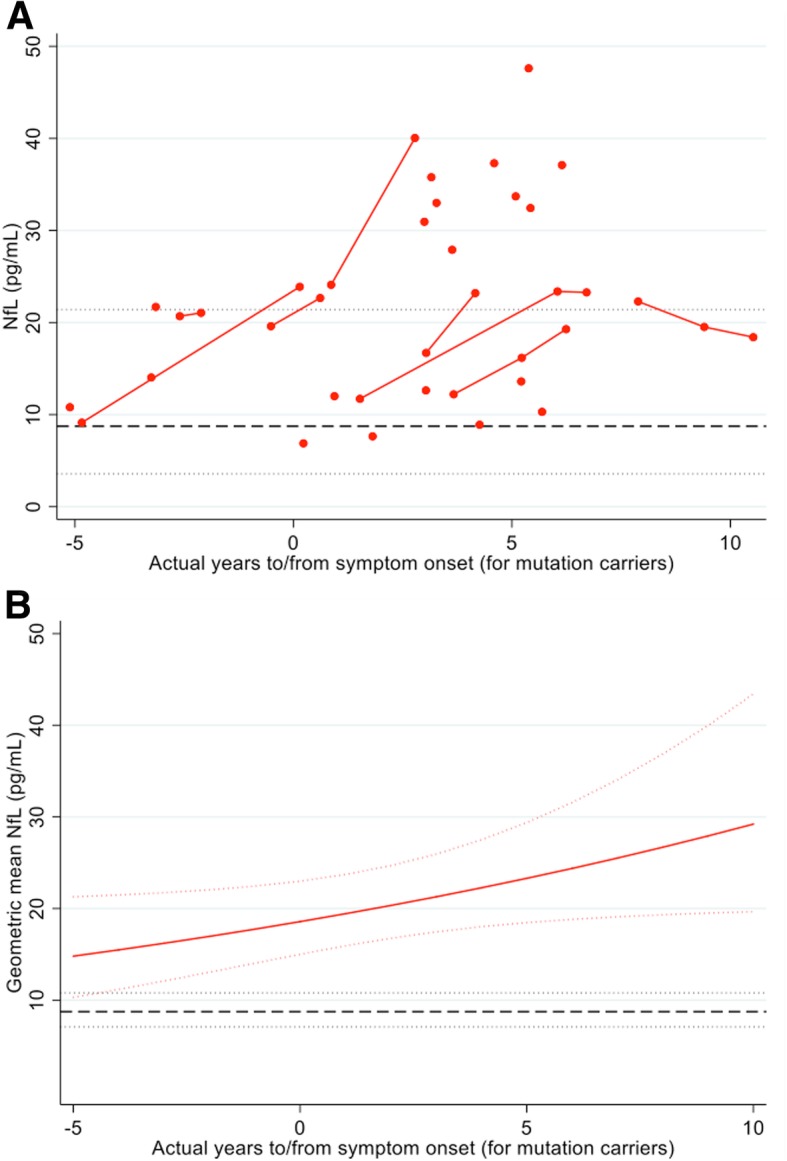
Serum NfL against AYO. **a** (In red) measured serum NfL concentration against AYO, for the 27 mutation carriers who have already developed symptoms (either before or during the study). Those measurements that belong to the same individual are connected by a line. No jitter has been applied. The black broken line represents the mean serum NfL concentration for non-carriers, with dotted lines for ± 1.96 SD, calculated on the log scale and back transformed. By showing ± 1.96 SD, we are indicating a reference range within which 95% of the observed values are expected to lie. **b** Geometric mean NfL modelled against AYO, for someone of average age (40.9 years) and sex (half male/female). The black broken line represents the geometric mean serum NfL concentration for non-carriers. Dotted lines indicate 95% confidence intervals (i.e. on repeated random sampling, 95% of CIs calculated in this way will include the true population geometric mean). AYO actual years to/from symptom onset. The geometric mean is the exponential of a mean calculated on the log scale

Estimated differences in NfL concentration between the two groups showed that NfL concentration was significantly increased (*p* = 0.045) in mutation carriers compared with non-carriers 15 years prior to expected symptom onset, after adjusting for age at visit and sex, and diverged progressively thereafter (Fig. 1b). Modelling NfL for the 27 mutation carriers with known AYO showed a progressive increase over the disease period that these data cover, consistent with the EYO-based analysis (Fig. 2).

Across all participants, adjusting for age at visit, sex, and EYO, there was no significant difference in NfL values between *PSEN1* carriers and *APP* carriers (*p* = 0.63). In *PSEN1*, there was no significant difference in NfL concentration between individuals with mutations pre and post-codon 200 (*p* = 0.67).

## Discussion

This study supports the utility of serum NfL as a potential biomarker of early neurodegeneration in presymptomatic AD. We extend previous findings by showing that not only is NfL concentration increased in presymptomatic FAD, but also this increase first occurs more than a decade before the onset of clinical symptoms and continues to rise thereafter.

Serum NfL has been associated with validated cognitive and structural imaging markers of disease severity in AD (Fig. 2) [1, 2, 7], including whole-brain volume, atrophy rates, and the MMSE. However, the initial change in NfL in our study appears to occur before these other measures are first thought to become abnormal [8, 9]. While markers of molecular pathology (i.e. CSF Aβ_1–42_ and amyloid PET) have been previously shown to change as early or earlier than markers of neurodegeneration [9–11], Aβ alone is not sufficient to cause symptomatic AD; therefore, downstream markers of neuronal loss are vital in helping identify those who are closest to symptom onset [12].

The comparatively early change in NfL compared to other validated markers of neurodegeneration, combined with the relative accessibility and non-invasiveness of a blood-based measure, suggests the potential utility of NfL as a screening measure in presymptomatic trials, and possibly even in the general population if disease-modifying treatments were to become available. However, our sample is relatively young, and it would therefore be important to assess how serum NfL varies between presymptomatic AD and healthy ageing in older individuals (i.e. the age range more relevant to sporadic AD) before any such more general use could be considered.

In mutation carriers, within-person longitudinal measurement of NfL generally demonstrates progressive increase with increasing follow-up time. There is, however, significant inter- and intra-individual variability, with some people exhibiting large changes over relatively short time intervals. Sample processing is unlikely to account for this variability as subject and QC samples were analysed in a consistent manner and QC CV between plates was less than 5%. NfL is a relatively non-specific marker of neuronal damage, and increases can be seen with neurodegeneration and also with neuroinflammation [1, 4, 13]. Coexistence of these pathological processes in FAD may be partly responsible for fluctuations seen in NfL concentration. Equally there may also be physiological factors that have an effect. Further investigation, and improved understanding, of confounding causes of alterations in NfL concentration is needed before this biomarker can be used in clinical practice.

EYO based on parental age at onset is a relatively reliable proxy measure of proximity to symptom onset [14]. However, EYO carries a degree of error, so using AYO when known may provide additional accuracy. As with the EYO-based model, analysis based on AYO shows progressive increase as individuals approach and then pass expected onset. The consistency between these models confirms that NfL is a marker of presymptomatic neurodegeneration and remains elevated after the onset of symptoms.

*PSEN1* carriers have been reported to have more widespread atrophy patterns in the early stages of disease progression than *APP* carriers [15]. However, after adjusting for age at visit, sex and disease stage (i.e. EYO), we found no difference in NfL concentration between *PSEN1* and *APP* carriers. Also, although *PSEN1* mutations located post-codon 200 often have more complex clinical phenotypes than those pre-codon 200, this did not translate to a difference in NfL.

Our study has limitations. The sample size was limited, owing primarily to the relative rarity of FAD mutations, and replication, especially of sub-group analyses, is required in larger cohorts. Given the sample size and the degree of fluctuation in NfL concentrations, it was not possible reliably to model rate of change over time within subjects. Also, due to the reliance on EYO, it is difficult to say with certainty the precise time point that NfL first increases. Further assessment of serum NfL in presymptomatic sporadic AD will also be important.

## Conclusions

We show that serum NfL is already increasing early in the presymptomatic phase of FAD and rises progressively thereafter. It will be important for the sources of intra-individual variability over time to be understood. Overall, however, serum NfL is a promising, easily accessible marker of early neurodegeneration in AD.

## Additional file

Additional file 1: **Table S1.** Participants’ family mutations. (DOCX 17 kb)

## Abbreviations

AD: Alzheimer’s disease
*APP*: Amyloid Precursor Protein
AYO: Actual years to onset
CDR: Clinical Dementia Rating Scale
EYO: Estimated years to onset
FAD: Familial Alzheimer’s disease
MMSE: Mini Mental State Examination
NfL: Neurofilament light
*PSEN1*: Presenilin 1
SD: Standard deviation
SOB: Sum of boxes
