# Supplementary material for: Longitudinal measurement of serum neurofilament light in presymptomatic familial Alzheimer’s disease
Source: Alzheimers Res Ther. 2019 Feb 20;11:19. doi: 10.1186/s13195-019-0472-5 (PMC6383280; doi:10.1186/s13195-019-0472-5)
Supplement: Supplementary file 1 — Table S1. Participants’ family mutations. (DOCX 17 kb) [file 13195_2019_472_MOESM1_ESM.docx]

Additional file: Table S1 Participants’ family mutations

| **Gene** | **Mutation** | **Number of individuals** |
| --- | --- | --- |
| APP | p.Val717Gly | 1 S |
|  | p.Val717Ile | 2 S, 3 AR |
|  | p.Val717Leu | 1 S, 2 AR |
|  | p. Thr719Asn | 1 AR |
| PS1 | Intron 4 | 3 AR |
|  | p.Tyr115His | 2 S, 1 AR |
|  | p.Tyr115Cys | 1 AR |
|  | p.Glu120Lys | 2 S |
|  | p.Ser132Arg | 1 AR |
|  | p.Met139Val | 2 S, 3 AR |
|  | p.Val142Ala | 1 S |
|  | p.Met146Ile | 1 S, 2AR |
|  | p.Leu171Pro | 1 S |
|  | p.Glu184Asp | 1 S, 6 AR |
|  | p.Ile202Phe | 4 AR |
|  | p.His214Tyr | 2 AR |
|  | p.Leu262Phe | 1 AR |
|  | p.Pro264Leu | 1 S |
|  | p.Arg269His | 1 AR |
|  | p.Arg278Ile | 2 AR |
|  | p.Glu280Gly | 5 S, 7 AR |
|  | ΔE9* | 1 S |

The number of individuals from families with each mutation is given, divided in to either symptomatic (S) or asymptomatic but at risk (AR). Details relating to how many at risk participants for each mutation were mutation carriers is not given to ensure it is not possible for any at risk individual to attempt to deduce their mutation status. * The exon 9 deletion (NM_000021.3:c.869-1G>T; p.Ser290Cys;Thr291_Ser319del) commonly referred to as ΔE9.
